# The linguistic validation of Russian version of Dutch four-dimensional symptoms questionnaire (4DSQ) for assessing distress, depression, anxiety and somatization in patients with borderline psychosomatic disorders

**DOI:** 10.1186/s13104-015-1766-8

**Published:** 2015-12-12

**Authors:** V. S. Arnautov, D. V. Reyhart, A. B. Smulevich, N. N. Yakhno, B. Terluin, E. K. Zakharova, A. V. Andryushchenko, V. A. Parfenov, M. V. Zamergrad, D. V. Romanov

**Affiliations:** I.M. Sechenov First Moscow Medical State University of Ministry of health of Russia, Moscow, 119991 Russia; Department of General Practice and Elderly Care Medicine, EMGO-Institute for Health and Care Research, VU University Medical Centre, Van der Boechorststraat 7, 1081 BT Amsterdam, The Netherlands; JSC Valenta Pharmaceuticals, Moscow, 119530 Russia

**Keywords:** Psychosomatic disorders, 4DSQ, Cross-cultural validation, Linguistic validation, Cognitive interview

## Abstract

**Background:**

The four-dimensional symptom questionnaire (4DSQ) is an originally Dutch self-report questionnaire that has been developed in primary care to distinguish non-specific general distress from depression, anxiety and somatization. In order to produce the appropriate translated Russian version the process of linguistic validation has been initiated. This process has been done according to the “Linguistic Validation Manual for Health Outcome Assessments” developed by MAPI institute.

**Objective:**

To produce the appropriate Russian version of the 4DSQ that is conceptually and linguistically equivalent to the original questionnaire.

**Methods:**

The original Dutch version of the 4DSQ was translated by one translator into Russian. The validated English version of the 4DSQ was translated by another translator into Russian without mutual consultation. The consensus version was created based on two translated versions. After that the back translation was performed to Dutch, some changes were implemented to the consensus Russian version and the second target version was developed based on these results. The second target version was sent to an appropriate group of reviewers. Based on their comments, the second target version was updated. After wards this version was tested in patients during cognitive interview. The study protocol was approved by the Independent Interdisciplinary Ethics Committee on Ethical Review for Clinical Studies, and in compliance with the Helsinki Declaration and ICH-GCP guidelines and local regulations. Enrolled patients provided written informed consent.

**Results:**

After the process of forward and backward translation, consultant and developer’s comments, clinicians and cognitive review the final version of Russian 4DSQ was developed for assessment of distress, depression, anxiety and somatization.

**Conclusion:**

The Russian 4DSQ as a result of translation procedures and cognitive interviews linguistically corresponds to the original Dutch 4DSQ and could be assessed in psychometric validation for the further using in general practice.

**Electronic supplementary material:**

The online version of this article (doi:10.1186/s13104-015-1766-8) contains supplementary material, which is available to authorized users.

## Background

The Russian language tools for the diagnoses of mental disorders in general practice are presented as scales that assess levels of anxiety and depression, but there are very few questionnaires for the differential assessment of non-specific reactions to stress and somatized mental disorders, including somatoform dysfunction of the autonomic nervous system. To identify such questionnaires, the foreign (non-Russian) literature was reviewed. The primary goal of the search we performed amongst international scales was to find the scale which includes assessment of multiple conditions (depression, anxiety, distress and somatization). The results of the search singled out the 4DSQ that was developed in 1996 by the Dutch general practitioner B. Terluin [4]. This questionnaire was developed to identify the clinical phenomena of distress and somatization and to distinguish these phenomena from depression and anxiety in primary health care settings; these phenomena are difficult to assess using other scales [2, 3]. The development of descriptors of the questionnaire was based on data from ten studies that were conducted in various primary care institutions in the Netherlands. The validity of the criteria was evaluated by comparing the 4DSQ scores with the clinical diagnoses of general practitioners and their opinions on the supposed somatizations and the standardized diagnoses of mental disorders.

The 4DSQ is a self-rated questionnaire for patients. The questionnaire consists of 50 items that assess symptom severities on subscales for distress, depression, anxiety and somatization within the previous 7-day period. The analysis of the responses allow for the determination of the nature of the predominant component in the structure of patients’ mental disorders [4]. The 4DSQ was clinically validated in the Netherlands, has passed an assessment of cross-cultural reliability in Poland and has shown good psychometric properties [5, 6, 7–9].

The brevity and simplicity of the questionnaire appears to be optimal for its application in the Russian population of patients with somatoform dysfunction of the autonomic nervous system; thus we decided to perform a Russian-language validation of the 4DSQ.

The purpose of this study was to validate the 4DSQ in Russia in a sample of patients with somatoform autonomic dysfunctions and verify its equivalence to the original version. There are always risks that after translation the exact meaning of some points may be lost and cultural differences may influence the interpretation of the questions. Therefore, there is a need to make sure that the Russian version measures the same as the original Dutch questionnaire.

## Methods

In order to produce an appropriately translated Russian version the process of linguistic validation has been used. This process has been done according to the “Linguistic Validation Manual for Health Outcome Assessments” developed by MAPI institute (http://www.mapigroup.com). The 4DSQ is available as a Dutch and English version (Additional file 1), as well as a number of other languages (see http://www.emgo.nl/researchtools/4DSQ.asp). The developer of the 4DSQ, Dr. Berend Terluin, took part in all steps of the process, except of those steps which are performed by qualified independent translators.

The purpose was to produce a Russian version of the questionnaire that is conceptually and linguistically equivalent to the source measure and allows data pooling and analysis/comparison across countries.

According to the “Linguistic Validation Manual for Health Outcome Assessments” the following steps for the linguistic validation from non-English language into another non-English language were performed:

- Forward translation of the original Dutch questionnaire and of the English version.
- Creating a consensus forward translation (version 1.0).
- Backward translation to Dutch.
- Creating a second consensus translation (version 2.0).
- Clinical review (version 2.1).
- Cognitive interviews.
- Proofreading (final version).

### Forward translations

Two qualified translators, both native Russian speakers, proficient in the source language and in English, living in the target country (Russia), with experience in the translation of medical and pharmaceutical documents, including the questionnaires for patients and who have been briefed on the purpose of the work. The translators were provided by professional translation agency “Roid”, based in Moscow, Russia (http://www.roid.ru).

The original version of the 4DSQ in Dutch was translated by one translator into Russian. The validated English version of the 4DSQ was translated by another translator into Russian without mutual consultation.

The validated English version already existed (previously adapted into English). In order to facilitate the process and improve international harmonization (in agreement with the recommendations of MAPI institute), it was decided that Russian translation should be based on both the original Dutch and validated English versions.

The consultant reviewed the two versions, compared them with the original, and established a Consensus version 1.0 in consultation with the translators.

The consultant reported (in English) the translation decision to developer and process participants. Based on this discussion, the Consensus language version 1.0 was created.

The role of the consultant was to perform quality control for the translation performed. The consultant was an independent specialist, medical writer, Contract Research Organization employee.

### Backward translation

It was agreed by all participants a priori that the developer of 4DSQ organized the backward translation of consensus Russian language version 1.0 into Dutch language in order to be in strictly compliance with MAPI recommendations [2012—Linguistic Validation Manual for Health Outcome Assessments (MAPI institute, new edition)] and thus to provide the high quality of review. A professional translation company “Translavic BV” (http://www.translavic.com) performed backward translation of Russian Consensus language version 1.0 of 4DSQ in Dutch for Berend Terluin to assess concordance between the back-translated version and the original Dutch version.

The developer compared the original version of 4DSQ and the backward translation into Dutch language of consensus target language version 1.0. For each item the developer analyzed the backward translation and determined whether it appropriately reflected the consensus target language version. All discrepancies were carefully examined, the developer described with consultant the findings and decided whether these discrepancies required modifications in the consensus target language version and suggested appropriate amendments. In order to facilitate the translation process the previously validated English version was used by consultant as the “mother” version instead of the original Dutch version.

During these procedures some critical points were discovered (see Table 1) and the target Russian language version 2.0 was developed. For example, in English version item 19 was translated as “Worry”, therefore it was translated in Russian as “Бecпoкoйcтвo”. During the process of backward translation this item was back-translated into “rusteloosheid” (Dutch), which means “restlessness” in English. The original item speaks of “piekeren” (Dutch).

**Table 1:** Questions in which differences of responses are identified in compared groups of patients

Question number	English version	Dutch version	Russian version	Recommendations of B. Terluin (author)
Somatization				
3	Fainting?	Flauw vallen?	Oбмopoки?	Translation in the Russian version fully complies with the English version
7	Palpitation?	Hartkloppingen?	Учaщeннoe cepдцeбиeниe?	Translation in the Russian version fully complies with the English version
11	Shortness of breath	Benauwdheid?	Oщyщeниe нexвaтки вoздyxa?	It is recommended to remove word “oщyщeниe (sensation)”, to make the question “more difficult” for a patient
Distress				
17	Feeling down or depressed?	Neerslachtigheid?	Плoxoe или пoдaвлeннoe нacтpoeниe?	It is recommended to remove word “плoxoe (down)”, to make the question “more difficult” for a patient
22	Lack of energy?	Lusteloosheid?	Упaдoк cил?	Translation in the Russian version fully complies with the English version
41	Did you easily become emotional?	Snel emotioneel?	Bac былo лeгкo взвoлнoвaть?	Translation in the Russian version fully complies with the English version
48	Did you ever have to do your best to put aside thoughts about any upsetting event (s)?	Moet u de afgelopen week weleens uw best doen om gedachten of herinneringen aan (een) aangrijpende gebeurtenis (sen) van u af te zetten?	Baм былo oчeнь тpyднo oтoгнaть oт ceбя нeпpиятныe мыcли o вoлнyющeм coбытии или coбытияx?	It is recommended to make emphasis on “нeпpиятныx coбытия (unpleasant events)”, so it will be “… oтoгнaть oт ceбя мыcли o нeпpиятнoм coбытии или coбытияx (put aside thoughts about unpleasant event or events)…”
Anxiety				
24	Anxiety or panic attacks?	Angst- of paniek-aanvallen?	Tpeвoгa или пpиcтyпы пaники?	Translation in the Russian version fully complies with the English version
43	Were you afraid to travel on buses, streetcars/trams, subways or train?	Bang om te reizen in bussen, treinen of trams?	Бoялиcь ли Bы eздить нa aвтoбycax, тpaмвaяx, мeтpo или пoeздax?	Translation in the Russian version fully complies with the English version

According to the developer, this word describes a situation in which someone has to think constantly of something, usually something that is bothering the person a lot. Actually, this is not really thinking (as a deliberate activity of someone), it is more like thoughts running through one’s head all the time (often in circles, as people say), and these thoughts are difficult to stop or to control. The person wants to stop this “piekeren” but nevertheless it goes on and on, especially when someone tries to relax or fall asleep.

Going further, it was decided, in agreement with the developer, to use two different translations for item 19 in Russian version of the scale:

1. “нaзoйливыe тpeвoжныe мыcли”—“intrusive restless thoughts”.
2. “нaзoйливыe нeпpиятныe мыcли”—“intrusive annoying thoughts”.

These translations were discussed during clinician’s review step.

### Clinician’s review

The target version 2.0 was sent to an appropriate group of reviewers including clinicians. The criteria for appropriateness were: scientific degree, clinical trials experience, specialist in therapeutic neurology or psychiatrist/or specialist from Healthcare Regulatory system/or specialist from clinical trials ethics expertise.

Neurologists and psychiatrists participation in clinicians review process was justified by the fact that therapeutical neurologists and not general practitioners in fact treat the patients with the described above symptoms. General practitioners (GP) would be provided with the instrument for the deep analysis of those patients in order to involve GPs more deeply in diagnosis and treatment after further psychometric validation of 4DSQ. Reviewers made comments, suggested changes in the translation, and returned it to the institute.

Created target version 2.1 was used for cognitive interview.

### Cognitive interviews

Ten patients with diagnosis “Disorders of autonomic nervous system” (4 women and 6 men) in I.M. Sechenov First Moscow Medical State University’s clinic at mean age of 35.4 ± 12.54 (Mean ± SD) years (the minimum age of 19 years, the maximum age of 54 years) were interviewed by the consultant Tatiana Pukhalskaya (MD, PhD, certified translator in English). The aim of this interview was to test the scale in patients group by means of asking them about questionnaire. The interview was structured in accordance with the protocol of cognitive interview.

The consultant tested the target version 2.1 on all subjects during individual in-depth, face-to-face interviews.

During each interview, the consultant first asked the respondent to complete the questionnaire.

After completion, the consultant reviewed each item of the target version 2.1 with the subject and asked whether the subject encountered any difficulty while responding.

The cognitive study was conducted during the period from July 2013 to December 2013.

If patient reported difficulties in any item of questionnaire completion, forward and backward translations were performed to analyze the origin of it.

It makes sense to note that four patients from the target population have paid their attention to item 21, considering the phrase “cмyтнoe чyвcтвo cтpaxa” (a vague feeling of fear), and offering to replace it with the words “нeoбъяcнимoe” or “нeocoзнaннoe” feeling of fear. However, it should be noted that the actual wording fully and the most adequately covers the translation of the validated English version of 4DSQ. Such difficulties have also occurred with items 28, 32, 36 and 38, but the actual wording fully and the most adequately covers the translation of the validated English version of 4DSQ:

Item 28—two patients did not understand the reference of the words “everything is meaningless”. The patients had decided that it refers to the treatment;

Item 32—three patients did not understand that the phrase “all this” refers to the previous questions;

Item 36—two patients did not understand the reference of the phrase “all it”, probably to the all the above mentioned;

Item 38—the word “thinking clearly” was not clear enough for two patients.

Based on the discussion of unclear items with the patients, consultant suggested that it seems appropriate to reconsider the wording of the one which was unclear to four patients: the word “дypнoтa” (feeling light-headed) could be translated as “пpeдoбмopoчнoe cocтoяниe”.

This recommendation was implemented into the final Russian version of 4DSQ.

## Results

Based on the results of forward and backward translation, consultant and developer’s comments, clinicians’ and cognitive review the final version of Russian 4DSQ was developed.

The further study for the psychometric validation based on linguistically validated Russian questionnaire was planned. In general, the translation process of the Russian version of the 4DSQ questionnaire development based on the Dutch questionnaire proved to be successful. We suppose that the scheme of linguistic validation that we used could be used for other languages as well.

## Discussion

After all steps of linguistic validation the Russian 4DSQ-scales have demonstrated the same linguistic structure as the original Dutch scales. Therefore, we can propose that this version could be used in general practice as a diagnostic tool after psychometric validation. Anyway there is a need of study of 4DSQ’s potential in the large population, especially in regions.

### Strengths and limitations

The main limitation is that this validation does not include psychometric validation which is required for the further scale recommendation in general practice.

Also the limitation of the study concerns the representativeness of the sample of the Clinic of Nervous Diseases of the First Moscow State Medical University (who participated in cognitive interview) for Russian speaking patients in general and small patients sample size. Russian speaking population is much differentiated in many ways, and we cannot entirely exclude the possibility that the study in some clinics in other regions would have different results.

When larger datasets in Russian speaking people will be available, future studies could provide analysis that is more detailed by more parameters and that could be the base for updated Russian version of the 4DSQ.

## Conclusions

The Russian 4DSQ as a result of translation procedures and cognitive interview linguistically corresponds to the original Dutch 4DSQ and could be used in further studies and general practice.

## Additional file

10.1186/s13104-015-1766-8 Four-Dimensional Symptom Questionnaire (4DSQ), English version, text revision 2010.
